# How and when perceived COVID-19 crisis strength impacts individuals' life satisfaction and sleep quality: A moderated mediation model

**DOI:** 10.3389/fpubh.2022.944942

**Published:** 2022-08-31

**Authors:** Yuanyuan Lan, Changlin Han, Xiaotong Liu, Qinqin Cao, Siyuan Chen, Yuhuan Xia

**Affiliations:** ^1^School of Business, Qingdao University, Qingdao, China; ^2^School of Economics and Management, Beijing Jiaotong University, Beijing, China; ^3^School of Innovation and Entrepreneurship, Shandong University, Qingdao, China

**Keywords:** perceived COVID-19 crisis strength, perceived risk of being infected, life satisfaction, sleep quality, trust in local government, mindfulness

## Abstract

The COVID-19 pandemic has caused millions of deaths, seriously hampering people's lives and their productivity. Drawing on social information processing theory, this research developed a moderated mediation model to investigate the influence of perceived COVID-19 crisis strength on individuals' well-being. The results from a sample of 441 suggest that individuals' perceived COVID-19 crisis strength indirectly affects their life satisfaction and sleep quality *via* their perceived risk of being infected. Moreover, both individuals' trust in local government and mindfulness trait can buffer the positive effect of perceived COVID-19 crisis strength on their perceived risk of being infected. At the same time, they also buffer the indirect impact of individuals' perceived COVID-19 crisis strength on life satisfaction and sleep quality through perceived risk of being infected. This research provides several practical implications for governments and individuals to mitigate the negative influences of the COVID-19 pandemic and help individuals boost life satisfaction and sleep quality.

## Introduction

The coronavirus disease that emerged in 2019 (COVID-19) has resulted in a devastating global event that has disrupted personal and work lives, caused a global economic slowdown, put a heavy strain on healthcare systems, and created a great deal of uncertainty for workers (1–3). The COVID-19 pandemic has lasted for more than 2 years. From the first known outbreak of COVID-19, the cumulative number of confirmed cases of infections has exceeded 513 million worldwide, including more than 6 million deaths. In China, people have experienced many COVID-19 waves, and recently the “highly mutated” Delta and Omicron variants resulted in another wave of infections. Overall, the COVID-19 outbreak has upended people's normal lives, causing great mental stress and tremendous public anxiety.

In the existing literature, numerous researchers have demonstrated the negative effects of the COVID-19 pandemic on the psychological and behavioral outcomes of individuals. For example, Trougakos et al. (4) demonstrated that COVID-19 health anxiety could increase individuals' emotion suppression, thereby adversely affecting their psychological need fulfillment. Unmet psychological needs can not only reduce individuals' goal progress and family engagement, but also trigger more somatic complaints in individuals. Research by Yoon et al. (5) showed that COVID-19 news consumption was positively related to increased uncertainty, which, in turn, negatively affects individuals' goal progress and creativity in the workplace. Lin et al. (6) found that the COVID-19 pandemic increased employees' job insecurity and further triggered employees' emotional exhaustion, organizational deviance, and saving behavior. Other studies have also tried to explore the factors that could mitigate the detrimental influences of the COVID-19 pandemic on individuals. For example, Chen et al. (1) found that individuals' proactive personality was associated with perceived strengths use, and thus their performance, resilience, and thriving will remain at a higher level than those with lower proactive motivation in the COVID-19 pandemic.

Despite the great progress in studying the COVID-19 pandemic, there still remain several unanswered questions. To begin with, research on the impact of perceived COVID-19 crisis strength is in its infancy. To a large extent, perceived COVID-19 crisis strength refers to an individual's judgment regarding COVID-19 severity (7). Existing studies primarily focus on the impact of the COVID-19 pandemic itself on individuals (8, 9), rather than the impact of their judgment of the severity of COVID-19 on individuals. Individuals' attitudes and subsequent behaviors are, to a large extent, directly determined by their judgment of what they are confronted with. Individuals' subsequent responses can vary because their perceived COVID-19 crisis strength is different. Therefore, it is necessary to further explore the influence of perceived COVID-19 crisis strength. Moreover, little is known about the underlying mechanism and potential boundary conditions by which individuals' perceived COVID-19 crisis strength affects their well-being. Well-being indicates a positive physical, mental, and social condition (10). Throughout the COVID-19 pandemic, people's well-being has been particularly vital for social stability. Although previous studies have examined the impact of perceived COVID-19 disruption on well-being (11, 12), it is unclear how and when perceived COVID-19 crisis strength predicts individuals' well-being.

Therefore, in this study, drawing upon social information processing theory, we developed a model to explore how individuals' perceived COVID-19 crisis strength affects their well-being. The core assumption of social information processing theory is that individuals view received social information as a crucial cue, which can significantly affect their attitudes, cognitions, and behaviors (13). In the current study, given that the COVID-19 pandemic is a stressful social event, we proposed that individuals' perceived COVID-19 crisis strength may increase their perceived risk of infection, which indicates a “subjective assessment of the probability of a specified type of accident happening and how concerned we are with such an event” (p.152) (14). When individuals perceive a high risk of COVID-19 infection, their well-being, such as life satisfaction and sleep quality, declines. Morgeson et al. (15) indicated that the strength of an event is considered from three dimensions: novelty, disruption, and criticality. In other words, the more novel, disruptive, and critical an event is, the more likely it is to influence individuals' recognitions and behaviors. Thus, when individuals perceive that the COVID-19 pandemic will be more severe, they may feel at high risk of infection, which is negatively related to their happiness and well-being—but positively related to their death distress (16, 17).

Furthermore, social information processing theory also shows that individual differences can alter the extent to which individuals interpret and respond to received social information (13). In this study, our attention focused on individuals' trust in local government and the personal trait of mindfulness, and we attempt to explore how these two factors moderate the relationship between individuals' perceived COVID-19 crisis strength and perceived risk of infection. Specifically, trust in local government means individuals believe the actions taken by local governments during the COVID-19 outbreak is effective and correct (18). Individuals who trust in local government are apt to believe that government could do a good job of environmental sanitizing, and take effective actions to protect citizens' lives, thus perceiving a low risk of being infected. However, when perceiving the same strength of the COVID-19 pandemic, individuals who do not trust local government may not believe the government is able to adopt effective COVID response policies, thus perceiving a high risk of being infected. In this study, we speculate that individuals' trust in local government may attenuate the positive relationship between perceived COVID-19 crisis strength and perceived risk of infection.

**Figure 1:**
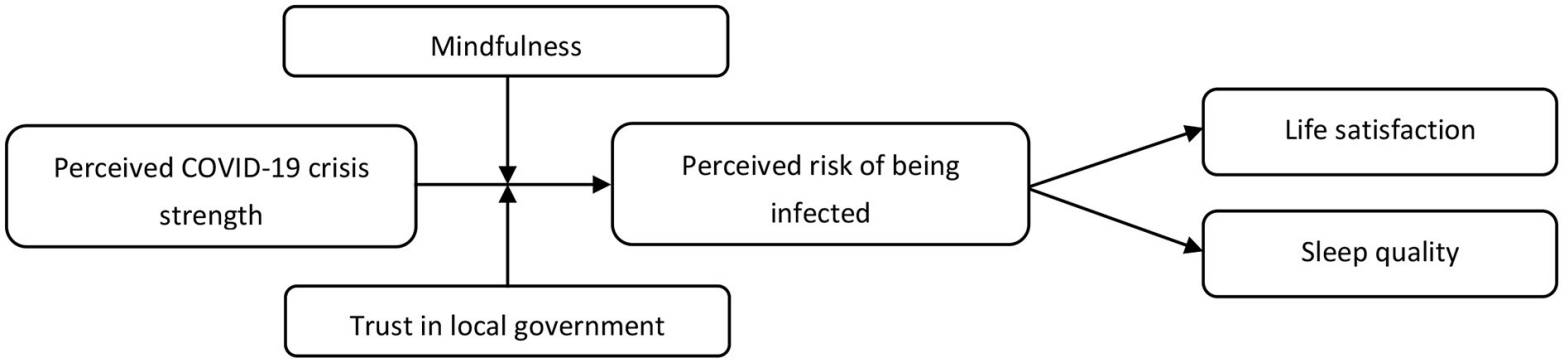
Conceptual model.

Mindfulness involves a non-judgment awareness and attention to the current moment (19), which refers to openness, awareness, and receptive attention (20). In other words, individuals with a higher level of mindfulness often purposely focus their attention on their ongoing and present experiences, as well as maintain a non-judgmental attitude (19, 21). Existing works suggest that mindfulness intervention is helpful for decreasing anxiety, depression, and emotional exhaustion during the COVID-19 pandemic (22), and is effective for relieving stress after COVID-19 lockdowns (23). Moreover, Zheng et al. (24) indicated that the interaction between COVID-19 stressors and mindfulness could affect sleep duration. Consistent with these works, we posit that, compared to individuals with low levels of mindfulness, those with high levels of mindfulness are less likely to perceive risk of being infected because they have the ability to fully experience the event without resorting to extremes either over-focusing on or inhibiting the experience. Therefore, mindfulness may buffer the positive effect of perceived COVID-19 crisis strength on perceived risk of infection. Figure 1 shows the theoretical model.

## Theoretical background and hypotheses

To understand the consequences of the COVID-19 pandemic, this research developed a moderated mediation model to explain the potential effects of individuals' perceived COVID-19 crisis strength on their well-being by using social information processing theory. Social information processing theory indicates that when individuals receive social information, they may interpret the information and form their own cognition, which in turn, shapes their attitudes and behaviors (13). Furthermore, social information processing theory also proposes that individual differences can constrain the process through which individuals respond to obtained social information (13). In the existing literature, this theory has been found suitable for exploring the factors that may influence individuals' well-being. For example, drawing upon social information processing theory, Zhang et al. (25) found that organizations' socially responsible human resource management practices can positively promote employees' perspective-taking and, subsequently, boost their well-being. Meanwhile, they also showed that employees' substantive attributions to socially responsible human resource management practices can magnify the positive effects; however, employees' symbolic attributions may reduce the positive effects. In the context of the COVID-19 pandemic, individuals use various cues to evaluate the risk of being infected, which in turn, affects their well-being. Indeed, scholars have characterized crises, such as the COVID-19 pandemic as social information that individuals obtain from the social environment (26, 27). Therefore, based on social information processing theory, this paper constructed a conceptual model to reveal how individuals' perceived COVID-19 crisis strength affects their well-being, including life satisfaction and sleep quality.

### Perceived COVID-19 crisis strength and perceived risk of being infected

The current studies have indicated that the strength of an event refers to its novelty, disruption, and criticality (15). Specifically, novelty means the extent to which an event is a new or unexpected phenomenon, or the degree to which the event differs from existing events, behaviors, and features (28, 29). The disruption involves a discontinuity in the external environment, in which the situation has changed (30). In other words, disruption means things do not continue the way they did prior to the extent (15). Moreover, the criticality reflects the extent to which an event is important, essential, or a priority to an entity (31). Morgeson et al. (15) pointed out that the more critical an event, the more likely it will to be seen as a salient event and the more likely it will require more attention and actions. The perceived risk of being infected refers to individuals' subjective assessment of the possibility of their being infected by COVID-19 and how concerned they are about the COVID-19 infection (14, 32). Individuals' perceived risk related to COVID-19 infection means not only the likelihood of experiencing the detrimental consequences caused by COVID-19 infection, but also their affective reactions to the COVID-19 infection, which may include worry or concern about their own safety (17, 32).

In this section, this paper argues that individuals' perceived COVID-19 crisis strength may be positively related to their perceived risk of being infected. In the context of the COVID-19 pandemic, *novelty* refers to individuals' perception that the COVID-19 is new and unexpected; *disruption* refers to the degree to which individuals perceive their existing tasks are interrupted by COVID-19; and *criticality* refers to whether individuals perceive their long-term development will be affected by COVID-19 (3, 6, 33). According to social information processing theory (13, 25), the obtained information may shape individuals' cognitions and perceptions. COVID-19 is a disease caused by the novel coronavirus which is different from previously known coronaviruses. Faced with this novelty, individuals may have limited knowledge about the procedures or guidelines to deal with such a crisis effectively (34). In terms of the disruption, the COVID-19 pandemic has changed the world, and the crisis seems to have put everyone's lives on hold (35). Specifically, to control the spread of COVID-19, many individuals have had to stay at home, and many enterprises or stores have been closed (36). Individuals' usual work and life activities have been highly disrupted by COVID-19. Moreover, the global economy and cross-border exchanges have stagnated. For example, the COVID-19 pandemic has had a dramatic impact or disruption on the tourism industry (37, 38). It has been more than two years since the COVID-19 outbreak first broke out, and it is still unknown when the pandemic will end. It seems to be possible that COVID-19 is likely to continue to have significant influences on individuals, and cause uncertainty about their futures (39).

Accordingly, when individuals glean information cues regarding the COVID-19 pandemic from the social environment, and view it as more novel, disruptive, and critical, they are less likely to have the confidence to cope with COVID-19 well. They will experience more changes in their usual life and feel they lose control of their future lives. Finally, they will view the COVID-19 pandemic as more serious, which may increase individuals' perceptions and fears regarding COVID-19 infection. In contrast, those who perceive the COVID-19 pandemic as less novel, disruptive, and critical, may have fewer concerns about this crisis, and feel that the way they live and work does not require too many changes, and their future development will not be heavily affected by COVID-19. Their perceptions and fears about COVID-19 infection will remain at a low level. Thus, we propose the following hypothesis:

**Hypothesis 1:** Individuals' perceived COVID-19 crisis strength is positively related to their perceived risk of being infected.

### The mediating role of perceived risk of being infected

After illustrating the positive relationship between the strength of individuals' perceived COVID-19 crisis and their perceived risk of being infected, this paper further explores the influences of individuals' perceived risk of COVID-19 infection on their well-being focusing particularly on two forms of individuals' well-being: life satisfaction and sleep quality. Life satisfaction is a form of subjective well-being that reflects individuals' cognitive assessment of whether or not they are satisfied with their life (40). Sleep quality, another form of well-being, refers to a subjective evaluation of their sleep experience that includes not only a sense of rest upon waking, but also the satisfaction with sleep (41, 42). Existing studies have investigated the factors that may influence individuals' life satisfaction and sleep quality. For example, scholars have shown that job satisfaction and core self-evaluation can be positively associated with individuals' life satisfaction (43, 44). Kuppens et al. (45) examined the effects of emotions on individuals' life satisfaction judgment and found that positive emotions were positively related to life satisfaction, while negative emotions were negatively related to life satisfaction. Thomsen et al. (46) indicated that individuals' rumination was significantly associated with poor subjective sleep quality. In turn, poor quality of sleep can lead to more psychological and physical health complaints and increased negative effects, such as anxiety, fatigue, and depression (42).

In this study, this paper argues that individuals perceived high risk of being infected may negatively affect their well-being, including life satisfaction and sleep quality. Social information processing theory indicates that individuals can process the obtained social information to form their cognitions and further develop their behaviors (13). Individuals' perceived high risk of infection is inherently a stressful and negative cognition. Previous studies have demonstrated that individuals rely on their emotional experiences to form subjective evaluations of well-being (45). Therefore, the stressful and negative cognition may trigger their lower level of well-being judgment. In addition, individuals' perceptions of being at risk of infection requires them to devote more personal effort to cope with such risks. Thus, the perceived high risk of COVID-19 infection may drain individuals' psychological and physical resources, ultimately leading to emotional exhaustion (32), which is a strong predictor of a lower level of well-being. Furthermore, existing studies have provided some empirical support for this argument. For example, Kwok et al. (47) indicated that perceived COVID-19 risk was positively related to individuals' anxiety, worry, and disruption of daily routines. Zhang et al. (48) showed that the perceived risk of COVID-19 infection may induce distress and reduce life satisfaction among working adults. Accordingly, we propose the following hypotheses:

**Hypothesis 2a:** Individuals' perceived risk of being infected is negatively related to their life satisfaction.

**Hypothesis 2b:** Individuals' perceived risk of being infected is negatively related to their sleep quality.

Integrating the discussion on hypotheses 1 and 2 and drawing upon social information processing theory, we further speculate that individuals' perceived COVID-19 crisis strength may affect their well-being including life satisfaction and sleep quality *via* their perceived high risk of being infected. Thus, we propose the following hypotheses:

**Hypothesis 3a:** Individuals' perceived risk of being infected mediates the relationship between perceived COVID-19 crisis strength and life satisfaction.

**Hypothesis 3b:** Individuals' perceived risk of being infected mediates the relationship between perceived COVID-19 crisis strength and sleep quality.

### The moderating roles of trust in local government and mindfulness

After revealing the mediation mechanism of individuals' perceived risk of being infected in the relationship between perceived strength of the COVID-19 pandemic and individuals' well-being, going a step further, this research further explores the boundary conditions that may constrain this indirect effect. Social information processing theory suggests that individual differences may affect the process of individuals' interpretation and response to the obtained social information (13, 25). Therefore, in this section, this paper mainly focuses on the moderating effects of individuals' trust in local government and individuals' mindfulness.

Specifically, individuals' trust in local government refers to the extent to which individuals believe the actions taken by the local government are effective in dealing with COVID-19 (18). During the outbreak of the COVID-19 pandemic, as front-line administrative units, local governments have a responsibility to prevent the spread of the COVID-19 pandemic (49). To effectively manage the COVID-19 pandemic, many local governments have formulated appropriate response policies and made reasonable adjustments, according to the immediate development of the crisis within their jurisdiction (50). In the context of the COVID-19 pandemic, individuals' trust in local governments may depend on their evaluation of the governments' coping capacity and performance (51). In other words, when individuals perceive that the measures made by the local government are strongly effective in preventing and stopping the spread of COVID-19, they may view the local government as credible and trustworthy (52). Existing studies have indicated that individuals' trust in the local government can decrease their perceptions of risk related to the COVID-19 crisis (53). Shanka and Menebo (18) also found that individuals' trust in the government is a strong predictor of their behaviors, and those who have more trust in local government are less likely to complain about the policies and measures, and more likely to be confident in dealing with COVID-19. Therefore, we propose the following hypothesis:

**Hypothesis 4:** Individuals' trust in local government moderates the relationship between perceived COVID-19 crisis strength and perceived risk of being infected, such that this relationship will be less positive when individuals' trust in local government is high.

In addition, this paper also explores the moderating effect of individuals' personal trait of mindfulness on the relationship between individuals' perceived COVID-19 strength and their perceived risk of infection. Mindfulness refers to receptive attention and awareness of present events and experiences (19, 54–56). Previous studies have demonstrated that mindfulness has a positive impact on human functioning, including attention, emotion, cognition, and behavior (19). In particular, the research on organizational behaviors has shown a positive association between mindfulness and improved workplace functioning (57). For example, the personal trait of mindfulness has been linked to higher job performance (58, 59) and citizenship behavior (54). Other studies have also demonstrated there are positive relationships between trait mindfulness and individuals' prosocial behavior and ethical behavior, as well as negative relationships between mindfulness and deviant behavior and counterproductive behavior (59, 60).

In this section, this research speculates that mindfulness may attenuate the positive relationship between perceived COVID-19 crisis strength and perceived risk of infection. Specifically, mindful individuals may be more aware and attentive when doing things, rather than automatically running through their tasks and activities (21, 61). In other words, mindfulness involves a process of decoupling, and mindful decoupling allows individuals to mentally step back from and observe present moment internal states and external events from a metacognitive perspective (54, 57, 62). That is, compared to those who are less mindful, mindful individuals will be more objective in observing current events, rather than immersing in the present experiences (62). Therefore, in the context of the COVID-19 pandemic, compared to those with a lower level of mindfulness, mindful individuals will be more likely to objectively analyze external situations they may face, and less likely to be concerned about COVID-19 infection. Existing research has provided some support for this argument. For example, Dillard and Meier (63) found that individuals with a higher level of mindfulness reported lower levels of stress, anxiety, worry, and negative emotions about COVID-19. In addition, they also noted that mindfulness was positively related to individuals' use of healthy coping strategies, such as seeking social support and positive reframing. Thus, we propose the following hypothesis:

**Hypothesis 5:** Individuals' mindfulness moderates the relationship between perceived COVID-19 crisis strength and perceived risk of being infected, such that this relationship will be less positive when mindfulness is high.

### Moderated mediation

Integrating the discussion for hypotheses 3, 4, and 5, we further propose that individuals' trust in local government and their level of trait mindfulness not only moderate the direct relationship between their perceived COVID-19 strength and perceived risk of being infected, but also the indirect relationship between their perceived COVID-19 strength and their well-being (i.e., life satisfaction and sleep quality) *via* perceived risk of being infected. This argument is consistent with Howell et al.'s (64) findings that mindfulness is positively associated with self-regulation of sleep and well-being. Accordingly, we propose the following hypotheses:

**Hypothesis 6:** Individuals' trust in local government moderates the indirect relationship between perceived COVID-19 crisis strength and (a) life satisfaction, and (b) sleep quality via perceived risk of being infected, such that these indirect effects will be weaker when trust in local government is high.

**Hypothesis 7:** Individuals' mindfulness moderates the indirect relationship between perceived COVID-19 crisis strength and (a) life satisfaction, and (b) sleep quality via perceived risk of being infected, such that this indirect effect will be weaker when mindfulness is high.

## Method

### Sample and procedure

The data for this research were collected from multiple subsidiaries of a large construction company. Most of these subsidiaries are located in Beijing, Tianjin, Henan province, and Guangdong province, and all of these cities and provinces had affected areas with confirmed COVID-19 cases when the data were collected in January 2022. With the help of these companies' human resource management departments, a total of 563 employees with management positions voluntarily participated in this survey. To obtain the truest thoughts of the participants, we promised all their responses would be anonymous, and all answers would be used for academic research only.

To improve the quality of the collected data, we removed responses that selected the same option for most questions (65). Moreover, given that a short response time meant that participants did not put enough effort into responding to surveys (66), we also removed respondents who took less than half the average time to answer the questionnaire^1^. In the end, 441 valid responses were obtained, yielding a 78.33% response rate. Among these valid samples, 61.22% were female, the average age was 32.10 years old (SD = 7.72), 58.96% of them held a bachelor's degree, and 9.52% held a master's degree or above.

### Measures

All English scales were translated into Chinese following the back-translation procedure (67), and we made minor modifications to the expression of some items to ensure all items were appropriate for our research context. All items were rated with a 7-point Likert scale, except the demographic variables.

*Perceived COVID-19 crisis strength*. Participants rated their perceived COVID-19 crisis strength using an 11-item scale developed by Liu et al. (7). A sample item was “This COVID-19 crisis causes me to stop and think about how to respond.” The Cronbach's α was 0.814. The anchors of this scale were a 7-point scale ranging from 1 = strongly disagree to 7 = strongly agree.

*Perceived risk of being infected*. Participants rated their perceived risk of being infected at work using an 8-item scale developed by Yildirim and Güler (68). A sample item was “Worry about oneself contracting COVID-19.” The Cronbach's α was 0.903. The 7-point scale ranged from 1 = negligible to 7 = very large.

*Life satisfaction*. Participants rated their life satisfaction using Cheung and Lucas's (40) 1-item scale: “Prior to any lifestyle changes due to COVID-19, in general, how satisfied are you with your life in the past week?” The anchors of this item were a 7-point scale ranging from 1 = very dissatisfied to 7 = very satisfied.

*Sleep quality*. Participants rated their life satisfaction using Lam et al.'s (69) 1-item scale: “How would you evaluate the quality of your past week's sleep?” The anchors of this item were a 7-point scale ranging from 1 = not very good to 7 = very good.

*Trust in government*. Participants rated trust in government with a 3-item scale developed by Shanka and Menebo (18). A sample item was “I think the government in my area is able to manage the COVID-19 pandemic properly.” The Cronbach's α was 0.895. The anchors of this scale were a 7-point scale ranging from 1 = strongly disagree to 7 = strongly agree.

*Mindfulness*. Participants rated their mindfulness with a 15-item scale developed by Brown and Ryan (21). A sample item was “I could be experiencing some emotion and not be conscious of it until some time later.” The Cronbach's α was 0.966. The anchors of this scale were a 7-point scale ranging from 1 = almost always to 7 = almost never.

*Control variables*. Prior works suggest that individuals' gender, age, and education level could influence their life satisfaction and sleep quality (70, 71). Thus, following previous research (72), we controlled participants' gender, age, and education level.

### Data analysis

Data were analyzed using SPSS version 25 and Mplus 8.3. We used the Chi-square degrees of freedom ratio (χ2/df), comparative fit index (CFI), Turker-Lewis index (TLI), root mean square error of approximation (RMSEA), and standardized root mean residual (SRMR) to examine the fit of the model to the data. When χ2/df is below 3, CFI and TLI are above 0.90, RMSEA is lower than 0.08, and SRMR is lower than 0.05, the results indicate a good fit. In addition, we conducted a structural equation model using maximum likelihood estimation with 5,000 bootstrap estimations to examine hypotheses 1 to 7.

## Results

### Discriminant and convergent validity

Mplus 8.3 was used to conduct a confirmatory factor analysis (CFA) to examine the discriminant validity of the key variables (i.e., perceived COVID-19 crisis strength, perceived risk of being infected, mindfulness, and trust in local government). Because both the scale of life satisfaction and sleep quality are a single item, this research did not include these two variables when conducting the CFA. As shown in Table 1, the four-factor model (χ2 = 1,318.371, df = 623, χ2/df = 2.116, CFI = 0.931, TLI = 0.926, RMSEA = 0.050, SRMR = 0.056) fit the data better than the other three models. The results reveal that our key variables have good discriminant validity.

**Table 1:** Confirmatory factor analysis.

**Model**	**χ^2^**	**df**	**χ^2^/df**	**CFI**	**TLI**	**RMSEA**	**SRMR**
Four-factor model: PCCS, PRBI, MIN, TILG	1318.371	623	2.116	0.931	0.926	0.050	0.056
Three-factor model: PCCS+PRBI, MIN, TILG	1715.814	626	2.741	0.891	0.884	0.063	0.068
Two-factor model: PCCS+PRBI+MIN, TILG	4062.829	628	6.469	0.658	0.637	0.111	0.159
One-factor model: PCCS+PRBI+MIN+TILG	4855.361	629	7.719	0.579	0.554	0.123	0.166

N = 441. PCCS, Perceived COVID-19 Crisis Strength; PRBI, Perceived risk of being infected; MIN, Mindfulness; TILG, Trust in local government. Same for following tables.

Factor loadings, average variance extracted (AVE), and reliabilities are presented in Table 2. The factor loadings of all items ranged from 0.538 to 0.920, higher than the threshold value of 0.5. The values of all composite reliability (CR) of the four variables ranged from 0.923 to 0.970, higher than the threshold value of 0.7. Moreover, the values of the AVE of the four variables ranged from 0.555 to 0.829, higher than the threshold value of 0.5. The results indicate our key variables have good convergent validity.

**Table 2:** Factor loadings, AVE, and reliabilities.

**Variables**	**Factor**	**Loadings**	**Cronbach's α**	**CR**	**AVE**
Perceived	PCCS1	0.825	0.814	0.931	0.555
COVID-19	PCCS2	0.739			
crisis	PCCS3	0.783			
strength	PCCS4	0.773			
	PCCS5	0.734			
	PCCS6	0.735			
	PCCS7	0.675			
	PCCS8	0.538			
	PCCS9	0.810			
	PCCS10	0.790			
	PCCS11	0.748			
Perceived	PRBI1	0.805	0.903	0.923	0.600
risk of being	PRBI2	0.768			
infected	PRBI3	0.729			
	PRBI4	0.704			
	PRBI5	0.791			
	PRBI6	0.788			
	PRBI7	0.804			
	PRBI8	0.803			
Mindfulness	MF1	0.865	0.966	0.970	0.681
	MF2	0.829			
	MF3	0.819			
	MF4	0.815			
	MF5	0.821			
	MF6	0.816			
	MF7	0.833			
	MF8	0.821			
	MF9	0.810			
	MF10	0.833			
	MF11	0.845			
	MF12	0.827			
	MF13	0.826			
	MF14	0.809			
	MF15	0.807			
Trust in local	TILG1	0.920	0.895	0.936	0.829
government	TILG2	0.910			
	TILG3	0.901			

N = 441.

### Descriptive statistics

Means, standard deviations, and correlations are presented in Table 3. The results showed that perceived COVID-19 crisis strength was positively related to perceived risk of being infected (r = 0.565, p < 0.01), and perceived risk of being infected was negatively related to life satisfaction (r = −0.428, *p* < 0.01) and sleep quality (r = −0.387, *p* < 0.01).

**Table 3:** Means, standard deviations, and correlations.

	**Mean**	**SD**	**1**	**2**	**3**	**4**	**5**	**6**	**7**	**8**	**9**
1. Gender	0.61	0.49	-								
2. Age	32.10	7.72	−0.208^**^	-							
3. Education level	2.83	0.98	0.225^**^	−0.032	-						
4. PCCS	4.06	0.89	−0.022	0.099^*^	−0.041	**(0.814)**					
5. PRBI	3.68	1.24	0.071	0.004	0.083	0.565^**^	**(0.903)**				
6. LS	4.59	1.53	−0.044	0.122^*^	−0.023	−0.405^**^	−0.428^**^	-			
7. SQ	4.74	1.49	−0.072	0.033	−0.072	−0.374^**^	−0.387^**^	0.542^**^	-		
8. MIN	4.15	1.22	0.115^*^	−0.051	−0.086	−0.198^**^	−0.164^**^	0.304^**^	0.233^**^	**(0.966)**	
9. TILG	5.06	1.41	0.061	−0.165^**^	−0.095^*^	−0.165^**^	−0.057	0.007	0.056	−0.121^*^	**(0.895)**

N = 441. LS, Life satisfaction; SQ, Sleep quality. Same for following tables. Internal consistent reliability (alpha) coefficients are shown along the diagonal in bold italics. Gender, 0 = male, 1 = female. Education, 1 = high school, 2 = associate degree, 3 = bachelor degree, 4 = master degree, 5 =Ph.D.

** p < 0.01,

* p < 0.05.

### Test of the direct effects

The results are presented in Table 4. The results showed that perceived COVID-19 crisis strength positively affected perceived risk of being infected (β = 0.664, 95% CI = [0.536, 0.793]), and perceived risk of being infected negatively affected life satisfaction (β = −0.347, 95% CI = [−0.467, −0.227]) and sleep quality (β = −0.291, 95% CI = [−0.409, −0.173]), supporting hypotheses 1, 2a, and 2b.

**Table 4:** Summary of direct, indirect, and moderate effects.

	**Estimates**	**S.E**.	**95% CI**	**Remarks**
Direct effects				
PCCS → PRBI	0.664	0.065	[0.536, 0.793]	Supported (H1)
PRBI → LS	−0.347	0.061	[−0.467, −0.227]	Supported (H2a)
PRBI → SQ	−0.291	0.06	[−0.409, −0.173]	Supported (H2b)
Indirect effects				
PCCS → PRBI → LS	−0.231	0.047	[−0.322, −0.139]	Supported (H3a)
PCCS → PRBI → SQ	−0.193	0.044	[−0.280, −0.106]	Supported (H3b)
Moderate effects				
TILG * PCCS → PRBI	−0.137	0.039	[−0.214, −0.060]	Supported (H4)
MIN * PCCS → PRBI	−0.155	0.046	[−0.246, −0.065]	Supported (H5)
Conditional indirect effects at values of TILG (PCCS → PRBI → LS)				
−1 SD (TILG)	−0.278	0.055	[−0.387, −0.170]	Supported (H6a)
+1 SD (TILG)	−0.183	0.043	[−0.268, −0.098]	
Difference	0.095	0.034	[0.029, 0.161]	
Conditional indirect effects at values of TILG (PCCS → PRBI → SQ)				
−1 SD (TILG)	−0.233	0.052	[−0.336, −0.130]	Supported (H6b)
+1 SD (TILG)	−0.153	0.040	[−0.232, −0.075]	
Difference	0.080	0.029	[0.023, 0.136]	
Conditional indirect effects at values of MIN (PCCS → PRBI → LS)				
−1 SD (MIN)	−0.285	0.057	[−0.396, −0.173]	Supported (H7a)
+1 SD (MIN)	−0.177	0.044	[−0.262, −0.091]	
Difference	0.108	0.04	[0.030, 0.185]	
Conditional indirect effects at values of MIN (PCCS → PRBI → SQ)				
−1 SD (MIN)	−0.238	0.053	[−0.343, −0.134]	Supported (H7b)
+1 SD (MIN)	−0.148	0.041	[−0.228, −0.068]	
Difference	0.090	0.033	[0.025, 0.155]	

N = 441. SE, standard error; CI, confidence interval. Values for quantitative moderators are the mean and plus/minus one SD from mean.

### Test of the indirect effects

The results in Table 4 also showed that the indirect effect of perceived risk of being infected in the relationship between perceived COVID-19 crisis strength and life satisfaction was −0.231 (95% CI = [−0.322, −0.139]), and the indirect effects of perceived risk of being infected in the relationship between perceived COVID-19 crisis strength and sleep quality was −0.193 (95% CI = [−0.280, −0.106]), respectively. Thus, hypotheses 3a and 3b were supported.

### Test of the moderating effects

**Figure 2:**
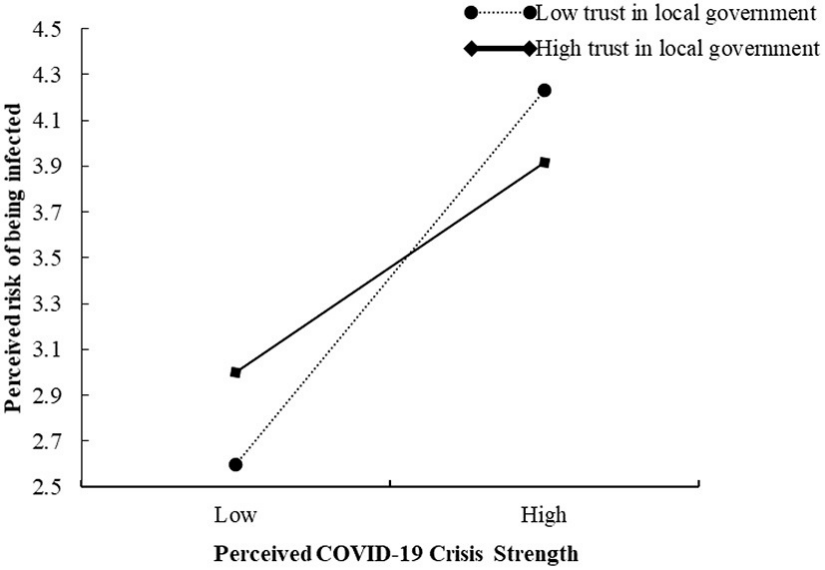
The moderating effect of trust in local government on the relationship between perceived COVID-19 crisis strength and perceived risk of being infected.

Moreover, the results in Table 4 also showed that the interaction between perceived COVID-19 crisis strength and trust in local government negatively affected perceived risk of being infected (β = −0.137, 95% CI = [−0.214, −0.060]), indicating that trust in local government negatively moderated the relationship between perceived COVID-19 crisis strength and perceived risk of being infected. To show the moderating effect more clearly, simple slopes for different levels of trust in local government were plotted (see Figure 2). Thus, Hypothesis 4 was supported.

**Figure 3:**
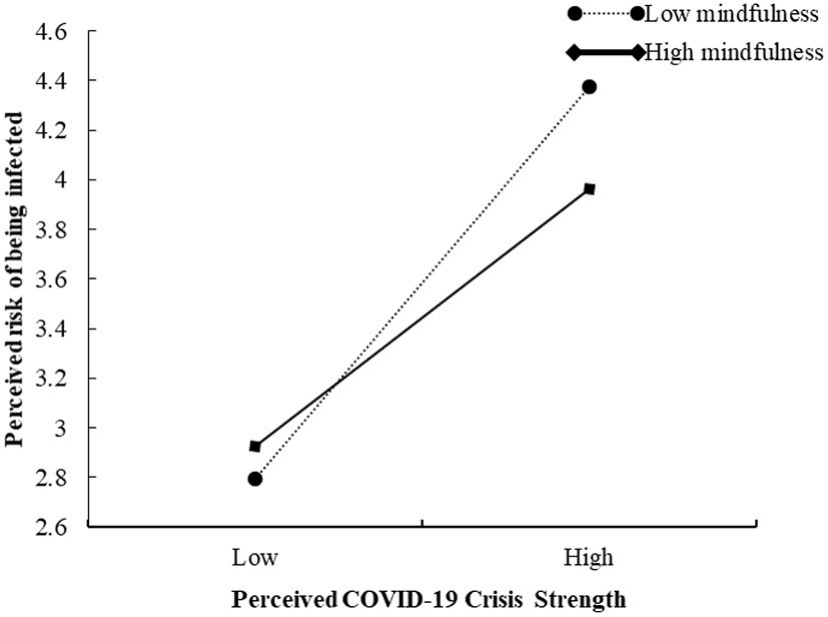
The moderating effect of mindfulness on the relationship between perceived COVID-19 crisis strength and perceived risk of being infected.

In addition, the interaction between perceived COVID-19 crisis strength and mindfulness negatively affected perceived risk of being infected (β = −0.155, 95% CI = [−0.246, −0.065]), indicating that mindfulness negatively moderated the relationship between perceived COVID-19 crisis strength and perceived risk of being infected. To show the moderating effect more clearly, simple slopes for different levels of mindfulness were plotted (see Figure 3). Thus, Hypothesis 5 was supported.

### Test of the moderated mediation effects

Furthermore, Table 4 also showed that perceived risk of being infected played a stronger mediating role in the relationship between perceived COVID-19 crisis strength and life satisfaction when individuals had a low level of trust in local government (i.e., conditional mediation effect = −0.278, 95% CI = [−0.387, −0.170]) vs. high (i.e., conditional mediation effect = −0.183, 95% CI = [−0.268, −0.098]), and the difference between the two indirect effects was 0.095 (95% CI = [0.029, 0.161]), supporting Hypothesis 6a.

Perceived risk of being infected played a stronger mediating role in the relationship between perceived COVID-19 crisis strength and sleep quality when individuals had a low level of trust in local government (i.e., conditional mediation effect = −0.233, 95% CI = [−0.336, −0.130]) vs. high (i.e., conditional mediation effect = −0.153, 95% CI = [−0.232, −0.075]), and the difference between the two indirect effects was 0.080 (95% CI = [0.023, 0.136]), supporting Hypothesis 6b.

Perceived risk of being infected played a stronger mediating role in the relationship between perceived COVID-19 crisis strength and life satisfaction when individuals had a low level of mindfulness (i.e., conditional mediation effect = −0.285, 95% CI = [−0.396, −0.173]) vs. high (i.e., conditional mediation effect = −0.177, 95% CI = [−0.262, −0.091]), and the difference between the two indirect effects was 0.108 (95% CI = [0.030, 0.185]), supporting Hypothesis 7a.

Perceived risk of being infected played a stronger mediating role in the relationship between perceived COVID-19 crisis strength and sleep quality when individuals had a low level of mindfulness (i.e., conditional mediation effect = −0.238, 95% CI = [−0.343, −0.134]) vs. high (i.e., conditional mediation effect = −0.148, 95% CI = [−0.228, −0.068]), and the difference between the two indirect effects was 0.090 (95% CI = [0.025, 0.155]), supporting Hypothesis 7b.

## Discussion

Drawing on social information processing theory, this paper developed a moderated mediation model to examine the influences of perceived COVID-19 crisis strength on individuals' well-being. The findings reveal that perceived COVID-19 crisis strength has a positive impact on perceived risk of infection, which in turn decreases life satisfaction and sleep quality. This finding not only validates previous studies' conclusions that, during the COVID-19 pandemic, individuals' well-being became worse (73) but also further substantiates the underlying mechanism by which individuals' perceived COVID-19 crisis strength affects their well-being. Moreover, both trust in local government and mindfulness negatively moderated the direct relationship between perceived COVID-19 crisis strength and perceived risk of being infected, as well as the indirect effects of perceived COVID-19 crisis strength on both life satisfaction and sleep quality *via* perceived risk of infection. Such findings are consistent with Ye and Lyu's (74) research, which suggests that risk perception is low for individuals with a high trust in government. Meanwhile, our findings also confirm Bossi et al.'s (23) and Matiz et al.'s (22) findings, who found that mindfulness-based training is beneficial for mitigating the negative impacts of the COVID-19 outbreak.

### Theoretical implications

This paper contributes to the literature in several ways. First, this research contributes to the COVID-19 literature by investigating individuals' life satisfaction and sleep quality in the context of the COVID-19 pandemic. Previous researchers have mainly focused on the influence of the COVID-19 pandemic on individuals' mental health such as anxiety (75), workplace behaviors such as work engagement (7), and tourists' responses such as health tourism intentions (76). Although some scholars have paid attention to individuals' well-being during the COVID-19 pandemic (77, 78), these researchers ignored individuals' life satisfaction and sleep quality, which have vital implications regarding their life and health. Our research not only responds to Lin et al.'s (6) call to further explores more outcomes of COVID-19, but also enriches the research on perceived COVID-19 crisis strength.

Second, this research contributes to the literature by testing how perceived COVID-19 crisis strength affects individuals' well-being from the information processing perspective. Previous research predominately investigated the COVID-19 pandemic from the perspective of event system theory (79); transactional theory of stress and coping (80); existence, relatedness, and growth theory (81), and so on. These studies focused on the intensity of the COVID-19 pandemic, or individuals' psychological responses to the COVID-19 pandemic. However, considering that the COVID-19 pandemic could be a kind of information cues from the social environment (82), how individuals process the social information they obtained also should not be overlooked. This research serves as a useful bridge to our understanding of the COVID-19 crisis with individuals' well-being, and provides a new perspective on how to reduce the negative impact of the COVID-19 pandemic.

Third, the current research contributes to the literature because it examines when perceived COVID-19 crisis strength decreases individuals' well-being in the context of the COVID-19 pandemic. Although previous empirical studies have discussed the boundary conditions for the COVID-19 pandemic's effects, these studies have been limited to the moderating role of job type (83), organizational tenure, health stressors (84), and so on, ignoring the positive influence of the government and mindfulness. Previous research (85) has indicated that when individuals have high trust in the government, they are more likely to engage in preventive measures. Such findings provide evidence for the buffering role of trust in local government. Moreover, evidence suggests mindfulness not only helps boost immunity, but also helps alleviate depression and anxiety (86). For individuals, therefore, mindfulness may be a potential boundary condition for the negative effects of the COVID-19 pandemic. This research enriches the nomological network of perceived COVID-19 crisis strength, thus contributing to a more complete understanding of how we can relieve the negative impacts of perceived COVID-19 crisis strength.

### Practical implications

This research also has several practical implications. First, this research confirmed that the perceived COVID-19 crisis strength is detrimental to individuals' life satisfaction and sleep quality. The COVID-19 pandemic has lasted for more than 2 years now. The facts have shown that, in the short term, this event cannot be prevented. However, individuals can mitigate the negative psychological effects of the COVID-19 pandemic by changing their minds. For example, individuals should pay more attention to positive news, such as that more and more research institutes are developing vaccines and medicines to fight against COVID-19, and many volunteers are currently fighting the pandemic. Moreover, the main reason individuals report poor life satisfaction and sleep quality is that they are afraid of being infected. Thus, to address these issues, individuals should take good protective measures, maintain good hygiene and health habits, and prepare for the sufficient necessities of life.

Second, the findings in this paper reveal that trust in local government could alleviate the negative effects of perceived COVID-19 crisis strength. Thus, it is vital to increase citizens' trust in local government. To begin with, governments should adopt authoritative and effective measures to fight against COVID-19. For example, the construction of a strong public health system must be accelerated, and nucleic acid detection capabilities and medical care capabilities must be enhanced. In addition, establishing a mechanism for observing and analyzing public opinion also helps increase citizens' trust. For example, government could rely on information systems to capture the events that cause public dissatisfaction, list the main events, analyze public opinion, and find the key points and requirements of the public. Furthermore, individuals should also trust government g so they can work together to win the anti-COVID war.

Third, in addition to trusting local government, this research also confirmed that individuals' mindfulness can help decrease the negative effects of perceived COVID-19 crisis strength. Although mindfulness is a kind of personality trait, individuals can gain high levels of mindfulness through training. For example, individuals can be trained in the following ways: mindful sitting meditation, body scan, mindful movement, 3-min breathing, lovingkindness meditation, focused attention, slowing down, and so on (87). To reduce the probability of contracting COVID-19, individuals can learn correct mindfulness practices through websites, books, and applications. In addition, individuals can try to connect with those who have high levels of mindfulness and learn some tips for increasing their mindfulness. In doing so, individuals will see a significant decrease in stress (19) and experience more well-being in their daily lives (88).

### Limitations and future research

As with previous research, this research has several limitations. First, this study used a cross-sectional design, which limits the ability to infer causality. Thus, future studies should adopt longitudinal designs to test the relationship between the focal variables in this paper. It would be interesting to see what happens to individuals' well-being as the COVID-19 crisis strength changes. Second, this research proposes and examines trust in local government and mindfulness as moderators that would mitigate the negative impacts of perceived COVID-19 crisis strength on individuals' life satisfaction and sleep quality. Yet, other moderators, such as family members, friends, and social factors should not be overlooked. For example, family members can provide support and comfort to individuals to help them override the negative effects caused by the COVID-19 pandemic. Third, the intensities of the COVID-19 pandemic and government response to COVID-19 vary around the world, so the influence of perceived COVID-19 crisis strength on individuals' well-being may also vary by country. Thus, future research could conduct cross-culture comparisons regarding the impacts of perceived COVID-19 crisis strength. Fourth, because all key variables measured in this study were perception based and the actual information people are attending to is not identified, future works should measure these variables with more objective methods.

## Conclusion

In sum, drawing on social information processing theory, this study investigated the effect of perceived COVID-19 crisis strength on individual well-being (i.e., life satisfaction and sleep quality). We further examined the potential mediating role of risk of being infected and the moderating roles of individuals' trust in local government and mindfulness in the relationship between perceived COVID-19 crisis strength and well-being. The results showed that individuals' perceived COVID-19 crisis strength can decrease their life satisfaction and sleep quality by strengthening the perceived risk of being infected. Furthermore, both individuals' trust in local government and mindfulness buffered the direct positive effect of perceived COVID-19 crisis strength on perceived risk of being infected, as well as the indirect effects of perceived COVID-19 crisis strength on both life satisfaction and sleep quality. Therefore, to promote individuals' life satisfaction and sleep quality, government is encouraged to adopt effective measures to fight against COVID-19 to increase people's trust, and individuals should undergo training to enhance their mindfulness.

## Data availability statement

The raw data supporting the conclusions of this article will be made available by the authors, without undue reservation.

## Ethics statement

This study was conducted according to the guidelines of the Declaration of Helsinki and approved by the Ethical Committee of School of Business, Qingdao University.

## Author contributions

YL: methodology, software, formal analysis, investigation, writing—original draft preparation, and supervision. YL and YX: conceptualization, validation. CH and XL: resources and data curation. CH, XL, QC, SC, and YX: writing—review and editing. All authors contributed to the article and approved the submitted version.

## Conflict of interest

The authors declare that the research was conducted in the absence of any commercial or financial relationships that could be construed as a potential conflict of interest.

## Publisher's note

All claims expressed in this article are solely those of the authors and do not necessarily represent those of their affiliated organizations, or those of the publisher, the editors and the reviewers. Any product that may be evaluated in this article, or claim that may be made by its manufacturer, is not guaranteed or endorsed by the publisher.

## Footnotes

^1^We tested our hypotheses without removing the responses that took less than half the average time to answer the questionnaire. The results showed that the exclusions did not affect the interpretation of the results.
